# Elucidation of the genetic basis underlying rooting ability in vegetatively propagated chrysanthemum

**DOI:** 10.1093/hr/uhaf289

**Published:** 2025-11-03

**Authors:** Xuefeng Zhang, Wei Sun, Jiangshuo Su, Ying Li, Jiafu Jiang, Zhiyong Guan, Fadi Chen, Weimin Fang, Fei Zhang

**Affiliations:** State Key Laboratory of Crop Genetics & Germplasm Enhancement and Utilization, College of Horticulture, Nanjing Agricultural University, Nanjing, Jiangsu 210095, China; Zhongshan Biological Breeding Laboratory, No.50 Zhongling Street, Nanjing, Jiangsu 210014, China; State Key Laboratory of Crop Genetics & Germplasm Enhancement and Utilization, College of Horticulture, Nanjing Agricultural University, Nanjing, Jiangsu 210095, China; Zhongshan Biological Breeding Laboratory, No.50 Zhongling Street, Nanjing, Jiangsu 210014, China; State Key Laboratory of Crop Genetics & Germplasm Enhancement and Utilization, College of Horticulture, Nanjing Agricultural University, Nanjing, Jiangsu 210095, China; Zhongshan Biological Breeding Laboratory, No.50 Zhongling Street, Nanjing, Jiangsu 210014, China; State Key Laboratory of Crop Genetics & Germplasm Enhancement and Utilization, College of Horticulture, Nanjing Agricultural University, Nanjing, Jiangsu 210095, China; Zhongshan Biological Breeding Laboratory, No.50 Zhongling Street, Nanjing, Jiangsu 210014, China; State Key Laboratory of Crop Genetics & Germplasm Enhancement and Utilization, College of Horticulture, Nanjing Agricultural University, Nanjing, Jiangsu 210095, China; Zhongshan Biological Breeding Laboratory, No.50 Zhongling Street, Nanjing, Jiangsu 210014, China; State Key Laboratory of Crop Genetics & Germplasm Enhancement and Utilization, College of Horticulture, Nanjing Agricultural University, Nanjing, Jiangsu 210095, China; Zhongshan Biological Breeding Laboratory, No.50 Zhongling Street, Nanjing, Jiangsu 210014, China; State Key Laboratory of Crop Genetics & Germplasm Enhancement and Utilization, College of Horticulture, Nanjing Agricultural University, Nanjing, Jiangsu 210095, China; Zhongshan Biological Breeding Laboratory, No.50 Zhongling Street, Nanjing, Jiangsu 210014, China; State Key Laboratory of Crop Genetics & Germplasm Enhancement and Utilization, College of Horticulture, Nanjing Agricultural University, Nanjing, Jiangsu 210095, China; Zhongshan Biological Breeding Laboratory, No.50 Zhongling Street, Nanjing, Jiangsu 210014, China; State Key Laboratory of Crop Genetics & Germplasm Enhancement and Utilization, College of Horticulture, Nanjing Agricultural University, Nanjing, Jiangsu 210095, China; Zhongshan Biological Breeding Laboratory, No.50 Zhongling Street, Nanjing, Jiangsu 210014, China

## Abstract

Chrysanthemum, a globally renowned economic crop, primarily relies on vegetative propagation methods such as cutting for commercial cultivation. However, certain varieties with exceptional ornamental qualities often encounter difficulties in widespread adoption due to poor rooting ability and suboptimal root quality. The genetic underpinnings of rooting ability in chrysanthemum cuttings have remained largely unexplored. This study marks a significant advancement in this field. By evaluating 11 rooting traits across a diverse panel of 188 chrysanthemum genotypes, we found that spray cut chrysanthemums exhibit superior rooting ability compared to other cultivated types and wild species. Selective sweep analysis identified 534 selected genomic regions potentially linked to rooting traits during the domestication and improvement of chrysanthemums. Genome-wide association studies (GWAS) conducted on four key rooting traits - total root length, root surface area, average root diameter, and number of roots, using multiple models discovered 71 significant SNPs and 98 candidate genes, including 21 differentially expressed genes identified via transcriptomic sequencing. A weighted gene co-expression network analysis further revealed two key modules (yellow and lightyellow) related to rooting traits. By integrating GWAS, transcriptomic data, and functional verification, we pinpointed the candidate gene *CmNRAMP3* as a negative regulator of rooting ability. These findings substantially enrich our understanding of the genetic mechanisms underlying rooting ability in chrysanthemum cuttings and provide a promising gene pool for improving rooting traits in future breeding programs.

## Introduction

The root system, a vital plant component, is critical to survival, development, and adaptation across diverse environmental conditions. Root system architecture (RSA), defined by the spatial distribution, density, length, and overall characteristics of a plant’s root system, exhibits remarkable plasticity and dynamic responses to environmental cues, enabling plants to adapt and mitigate growth constraints [21]. Given its functional significance, RSA has become a critical focus for breeders seeking to improve plant resilience and vigor. Chrysanthemum (*Chrysanthemum morifolium*), a perennial herb in the Asteraceae family, has major commercial value in ornamental, medicinal, and edible domains [40]. In commercial production, vegetative propagation through stem cuttings represents the primary mode of plant multiplication. The success of this process heavily depends on the rapid root initiation and robust root system, which determine propagation efficiency and subsequent plant performance [54]. However, significant challenges persist in chrysanthemum production, particularly with certain cultivars demonstrating poor rooting competence, suboptimal root system quality, delayed seedling establishment, and elevated post-transplant mortality rates. These issues highlight the need to optimize root system development for healthy, vigorous propagules. Consequently, elucidating the genetic mechanisms of adventitious root formation and identifying key regulatory genes for rooting capacity are priority research areas in chrysanthemum biology, providing a basis for molecular breeding of superior cultivars.

Root development is regulated by a complex network of regulatory factors, including plant hormone signaling (e.g. auxin, cytokinin, and abscisic acid), hormone transport, transcription factors, nutrient availability, and biosynthetic pathways. In *Arabidopsis*, abscisic acid inhibits auxin-mediated primary root elongation via NRP-dependent PIN2 vacuole degradation [46], while the WOX-ARF transcription complex mediates the formation of distinct root types [52]. In maize, the peroxidase gene *PRX1* enhances drought resistance by promoting root development and lignification [51]. While much progress has been made in field crops, recent studies in ornamental plants have also begun to uncover key regulators of root development. In carnation, transcriptome profiling revealed genes involved in hormone signaling, cell-wall remodeling, and energy metabolism during root development [41]. In rose, the phosphoinositide phosphatase gene *SAC9* significantly influences adventitious root formation, enabling the development of a KASP marker for marker-assisted selection of high rooting ability [42]. In chrysanthemums, genetic studies remain limited, with most research focusing on environmental and exogenous factors. Notably, the MADS transcription factor *CmANR1* promotes adventitious root formation by activating the auxin-transport gene *CmPIN2*, whereas *CmBT1* suppresses this process by inhibiting *CmANR1*-mediated development [11, 36]. Identifying additional key genes is crucial for advancing molecular breeding and improving propagation efficiency.

Rooting ability is a composite trait involving multiple root growth and RSA characteristics, such as root length, number, and area. Multiple genes govern these traits, most of which have minor effects, each contributing relatively little individually. Understanding the genetic basis of these complex quantitative and polygenic characteristics is crucial for advancing chrysanthemum breeding programs. Due to their complexity, traditional research techniques often face significant limitations. In contrast, single nucleotide polymorphism (SNP) markers, with high resolution, broad genome coverage, and genetic stability, offer a more appropriate approach for studying the genetic basis of rooting ability. Genome-wide association studies (GWAS) identify trait-associated genes by exploiting linkage disequilibrium between phenotypes and genetic variants [7, 38] and have been widely applied to root systems in plants. For instance, Sanchez *et al.* [32] detected one SNP associated with root-related gene expression in maize seedlings. Xu *et al.* [47] used a 90 K SNP chip-based GWAS to identify 24 SNPs for rice root traits, and sequence analysis revealed that the candidate gene *LOC_S111810g04* carried nonsynonymous mutations potentially affecting root number. Hu *et al.* [14] identified 19 candidate genes significantly associated with cotton lateral root number, confirming the pivotal roles of *FLA12*, *WRKY29*, and *RBOHA*. Such advancements have significantly enhanced our understanding of the genetic basis underlying the rooting capacity of plants.

GWAS research in chrysanthemums has commenced more recently, and to date, there are no reports specifically focused on rooting ability traits. This study assessed 11 RSA traits related to rooting ability in a diverse panel of 188 chrysanthemum accessions, encompassing various cultivated types and wild species, over two years. It successfully identified genomic regions under artificial selection for rooting capacity during chrysanthemum breeding. By integrating GWAS with transcriptome analysis, the study explored genomic regions and candidate genes associated with rooting ability, providing a foundation for future molecular breeding efforts. These findings offer novel insights into the genetic mechanisms underlying rooting traits in chrysanthemum cuttings and may serve as a valuable reference for improving other vegetatively propagated crops.

## Results

### Phenotypic performance

**Figure 1:**
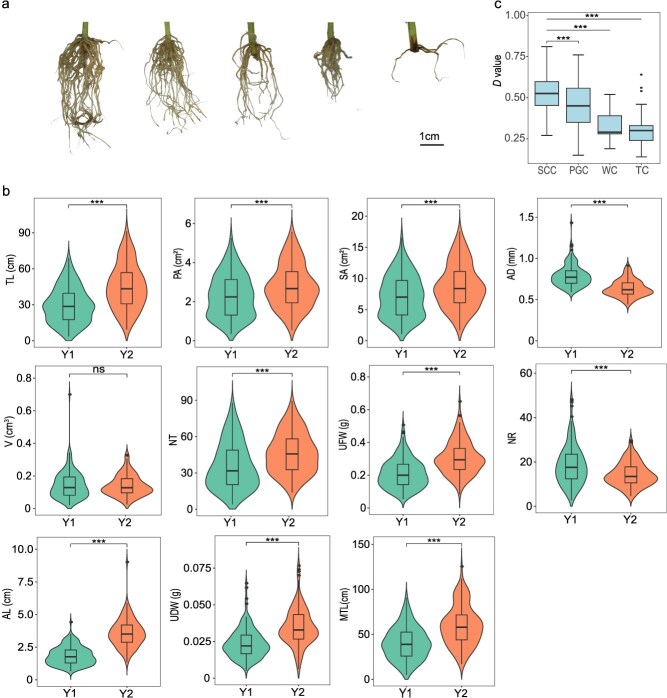
Phenotypic analysis of rooting traits in chrysanthemum accessions. (a) Root morphology of the representative chrysanthemum genotypes after 20 d of cutting. From left to right, the accessions are ‘Jinling Meiguijin’, ‘Nannong Huanglongyu’, ‘Nannong Bingqilin’, ‘Baipenghua’, and ‘Jiumiting’. Scale bar: 1 cm. (b) Violin plots showing the distribution of rooting traits across two years (Y1 and Y2). (c) Box plot of the *D* value of different chrysanthemum cultivated types. ^***^ indicates a significant difference at *P* < 0.001, and ‘ns’ denotes no significant difference (Student’s *t*-test).

The rooting ability of chrysanthemum cuttings exhibited significant variation among different genotypes (Figs 1a and b and S1). Descriptive statistical analysis showed considerable variation in 11 rooting traits across 188 chrysanthemum genotypes, with the coefficient of variation ranging from 21.15% (average root diameter; AD) to 67.16% (number of root tips; NT) (Table 1), highlighting a solid genetic basis for further analysis. Except for AD, average root length (AL), and underground dry weight (UDW) displaying significantly skewed distributions, most traits followed a normal distribution, with consistent trends observed over the two years. Correlation analysis revealed that most rooting traits were highly and significantly correlated across both years (Fig. S2). While AD showed a highly significant negative correlation with total root length (TL), number of root tips (NT), AL, and maximum total root length (MTL), other traits exhibited significant positive correlations. Notably, the correlation coefficients between TL and projected root area (PA), as well as SA and root volume (V), exceeded 0.9 in both years. Most traits, except for UDW, exhibited broad-sense heritability (*H^2^*) values exceeding 0.5, with V showing the highest at 0.68, indicating that root traits are relatively stable and heritable.

**Table 1:** Descriptive statistics for 11 rooting traits of 188 chrysanthemums.

**Trait**	**Year**	**Min**	**Max**	**Mean**	**CV *(*%)**	**SD**	**Skew.**	**Kurt.**	*H^2^*
TL/cm	Y1	0.30	104.29	30.61	57.28	17.53	0.65	0.16	0.62
	Y2	0.19	131.65	45.61	49.69	22.66	0.57	0.16	
PA/cm^2^	Y1	0.02	12.89	2.35	57.22	1.35	1.11	3.97	0.64
	Y2	0.01	8.64	2.82	48.92	1.38	0.60	0.19	
SA/cm^2^	Y1	0.05	29.78	7.34	55.12	4.05	0.64	0.32	0.64
	Y2	0.04	27.14	8.85	48.93	4.33	0.60	0.19	
AD/mm	Y1	0.06	2.30	0.79	22.64	0.18	1.61	7.86	0.55
	Y2	0.32	1.36	0.64	21.15	0.14	0.85	1.41	
V/cm^3^	Y1	0.00	0.68	0.15	62.07	0.09	1.19	2.46	0.68
	Y2	0.00	0.60	0.14	56.29	0.08	1.15	2.13	
NT	Y1	2.00	165.00	37.04	65.43	24.24	1.05	0.93	0.55
	Y2	2.00	132.00	46.71	46.82	21.87	0.68	0.35	
UFW/g	Y1	0.03	2.00	0.21	51.85	0.11	2.40	25.69	0.58
	Y2	0.05	1.09	0.31	42.23	0.13	1.01	1.59	
NR	Y1	1.00	86.00	19.04	55.04	10.48	1.18	2.24	0.55
	Y2	1.00	60.00	14.58	48.07	7.01	0.94	1.65	
AL/cm	Y1	0.01	24.67	1.84	67.16	1.24	4.51	60.57	0.52
	Y2	0.01	26.14	3.54	61.95	2.19	2.89	16.49	
UDW/g	Y1	0.00	0.23	0.02	56.90	0.01	6.13	80.75	0.37
	Y2	0.00	0.18	0.04	46.79	0.02	2.86	18.83	
MTL/cm	Y1	1.90	104.30	41.20	46.21	19.04	0.53	0.05	0.60
	Y2	2.95	131.65	59.93	39.74	23.82	0.54	−0.04	

Note: SD, standard deviation; CV, coefficient of variation; Skew., skewness; Kurt., kurtosis.

### Comprehensive evaluation of rooting capacity

Principal component analysis (PCA) was performed on the best linear unbiased estimations (BLUEs) of 11 rooting traits (Table S3). The first principal component (PC1) explained 64.77% of the variance, primarily capturing root area and length traits such as SA, PA, and TL. Notably, SA, measured by the WinRHIZO root image analysis system, was more effective at distinguishing small roots than PA. PC2 accounted for 16.76% of the variance and was dominated by AD, representing root system thickness. PC3 contributed 9.76% of the variance, with AL and NR making up the largest proportion, reflecting root number, and AL serving as a derivative of TL and NR. Therefore, TL, SA, AD, and NR are identified as key traits for chrysanthemum rooting. Furthermore, the membership function was applied to calculate the comprehensive indicator value (*CI*) from the PCA results, yielding membership function values (*U*) and comprehensive rooting ability scores (*D*) for assessing the rooting ability of chrysanthemum cuttings. ‘Nannong Lvyu’ exhibited the highest *D* value of 0.81, while ‘Quanxiang Huolong’ showed the weakest rooting ability with a *D* value of 0.14 (Table S1). A comparative analysis of *D* values across wild species and different cultivated types revealed distinct rooting capabilities. Wild chrysanthemum species (WC) had an average *D* value of 0.32 (ranging from 0.19 to 0.52), traditional chrysanthemums (TC) averaged 0.30 (with a range of 0.14 to 0.64), spray cut chrysanthemums (SCC) had the highest average *D* value of 0.53 (ranging from 0.27 to 0.81), and potted and groundcover chrysanthemums (PGC) averaged 0.44 (varying from 0.15 to 0.76). In summary, SCC demonstrated the highest rooting ability, followed by PGC, WC, and TC (Fig. 1c).

### Genetic structure

**Figure 2:**
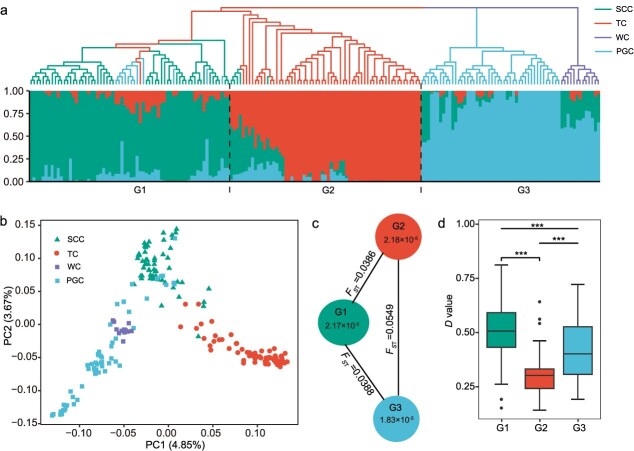
Population genomic analyses of 188 chrysanthemum accessions. (a) Combined display of a phylogenetic tree (top; based on whole-genome SNPs) and population structure analysis (bottom; *K* = 3). Individuals are ordered identically in both panels, and dashed lines delimit the three genetic subgroups (G1–G3). (b) Principal component analysis (PCA) of different cultivated germplasm categories, showing PC1 and PC2 (variance explained in parentheses). (c) Genetic diversity and population differentiation among the three subgroups. Values inside the circles represent nucleotide diversity (π) for each subgroup, and values on the connecting lines indicate pairwise population differentiation (*F*_ST_) between subgroups. (d) Box plot of *D* values assessing rooting ability in different subgroups. Asterisks denote statistically significant differences as analyzed by Student’s *t*-test (^***^  *P* < 0.001).

We conducted phylogenetic tree construction, population structure analysis, and principal component analysis (Fig. 2a and b). The accessions were classified into three main subgroups, G1, G2, and G3, respectively. These subgroups essentially correspond to chrysanthemum types with differing rooting abilities. G1 consisted of 70 accessions, including 12 from PGC, one from TC, and the remainder from SCC, with an average rooting ability *D* value of 0.51. G2 comprised 59 accessions, nearly all of which were TC, except for ‘Posuo Nufang’, and had an average rooting ability *D* value of 0.30. G3 comprised 13 WC and 46 PGC accessions, with an average rooting ability *D* value of 0.41. Pairwise *F_ST_* comparisons revealed significant genetic divergence among the three subgroups, with the greatest differentiation observed between G2 and G3 (*F*_ST_ = 0.0549) and the smallest between G1 and G2 (*F*_ST_ = 0.0386) (Fig. 2c). A notable reduction in genome-wide diversity was observed in G1 (π =2.17 × 10^−5^) and G3 (π =1.82 × 10^−5^) compared to G2 (π =2.18 × 10^−5^) (*P* < 0.01, Figs 2d and S3), reflecting the cumulative effects of breeding bottlenecks and directional selection associated with the distinct domestication histories of these cultivated types.

### Genome-wide selective sweeps

**Figure 3:**
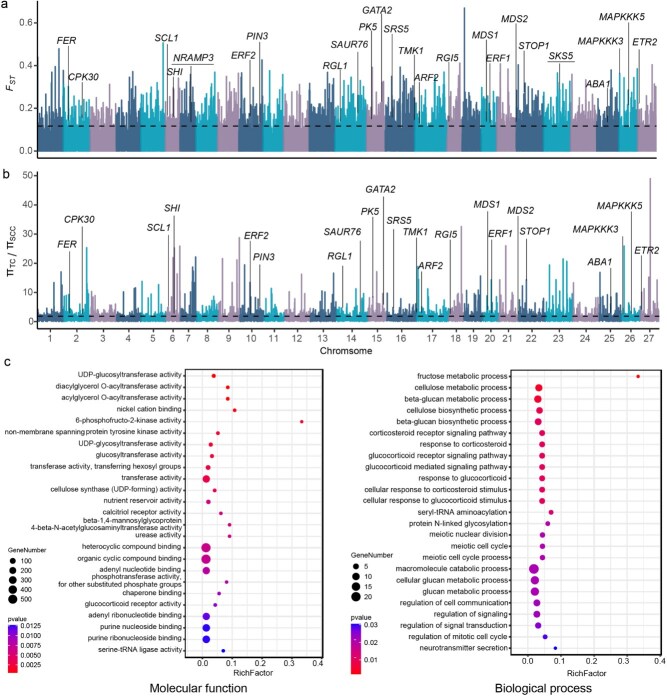
Analysis of genome-wide selective sweeps associated with rooting traits in chrysanthemum. (a, b) Selective sweep regions identified by *F_ST_* between SCC and TC populations, as well as by the π ratios (π_TC_/π_SCC_). The horizontal lines represent the threshold values corresponding to the top 5% of *F_ST_* and π ratios. Candidate genes with known associations to rooting traits in other species are labeled, whereas two GWAS-identified genes co-localized with selective sweep regions are underlined. (c) GO enrichment analysis of genes in the selective sweep regions.

A primary goal of modern chrysanthemum breeding is to attain faster rooting speeds and develop more robust root systems. Compared to traditional chrysanthemums, contemporary commercial cut chrysanthemums exhibit superior rooting abilities, likely resulting from artificial selection during breeding improvement processes. To identify potential selective sweeps associated with rooting traits, we analyzed high-quality SNPs obtained via genotyping-by-sequencing (GBS) and compared two groups of chrysanthemums with contrasting rooting performance (TC vs. SCC). We calculated genetic differentiation (*F_ST_*) between groups to capture divergence in allele frequencies, and assessed within-group nucleotide diversity (π) to evaluate reductions in genetic diversity, both of which are indicative of selective sweeps. Using top-5% thresholds for both *F_ST_* and π ratio (π_TC_/π_SCC_) distributions, we identified 3473 non-overlapping *F_ST_* selection intervals covering approximately 8.29% of the genome (Table S4; Fig. 3a) and 3086 π ratio intervals covering about 5.69% (Table S5; Fig. 3b). Integrating both approaches yielded 534 high-confidence selection intervals, totaling 76.93 Mb and distributed across all 27 chromosomes, with Chromosome 16 showing the most extensive coverage (Fig. S4). These selected regions encompass 1639 genes (Table S6). To further explore their potential functional roles, Gene Ontology (GO) enrichment analysis was conducted at the molecular function and biological process levels (Fig. 3c). At the molecular function level, the genes were enriched in pathways related to nonmembrane-spanning protein tyrosine kinase activity, transferase activity, cellulose synthase activity, and compound binding. At the biological processes level, the genes were associated with cellulose biosynthetic and metabolism, glucan metabolism, and signal transduction regulation. Many genes with orthologs involved in root growth and development have been identified across various species (Fig. 3a and b). For example, *evm.model.scaffold_797.35* (orthologous to *Arabidopsis RGL1*) modulates root growth through gibberellin signaling in *Populus* [3]; *evm.model.scaffold_772.60* (orthologous to *Arabidopsis TMK1*) is essential for root gravitropism by facilitating auxin gradient formation in *Arabidopsis* [44]; *evm.model.scaffold_1711.96* (orthologous to *Arabidopsis SRS5*) negatively regulates lateral root formation in *Arabidopsis* [50]; *evm.model.scaffold_2599.54* (orthologous to *Arabidopsis ETR2*) participates in ethylene signaling and modulates root development by interacting with auxin pathways in *Arabidopsis* [31]. These genes illustrate the conserved genetic mechanisms—spanning hormonal pathways such as gibberellin, auxin, ethylene, and abscisic acid—that regulate root growth across diverse plant species.

### GWAS for rooting traits

**Figure 4:**
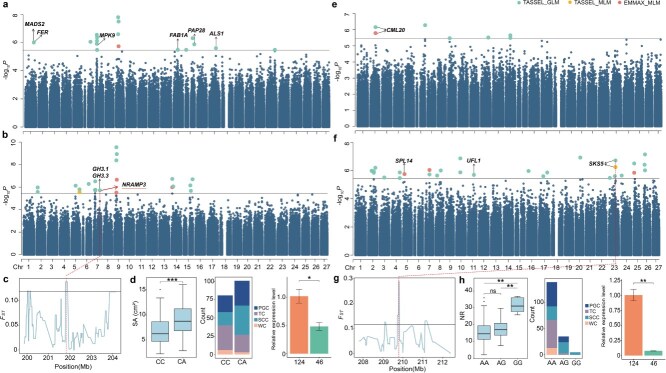
GWAS and candidate genes identification for rooting traits in chrysanthemum. Candidate genes of interest associated with rooting traits are highlighted above their corresponding SNPs. (a, b, e, f) Manhattan plots of GWAS results for TL (a), SA (b), AD (e), and NR (f) with the significance threshold by the line (-log_10_*P* = 5.47). (c, g) *F_ST_* selective signals for *NRAMP3* associated with SA (c) and for *SKS5* associated with NR (g), with dashed lines indicating the overlap of GWAS signals with selective sweeps. (d) Haplotype analysis and distribution of SA for different cultivated types at Chr7_201791696*,* along with a comparison of *NRAMP3* expression levels between accessions with weak (124) and strong [45] rooting abilities using qRT-PCR. (h) Haplotype analysis and distribution of NR for different cultivation types at Chr23_209923210, along with a comparison of *SKS5* expression levels between accessions with weak (124) and strong [45] rooting abilities. Asterisks indicate significant differences as analyzed by Student’s *t*-test (^***^  *P* < 0.001, ^**^P < 0.01, and ^*^P < 0.05). ‘ns’ indicates no significant difference.

We perform GWAS on four key rooting traits (TL, SA, AD, and NR) using the GLM and MLM models in TASSEL and the MLM model in EMMAX. A total of 71 SNPs significantly associated with rooting traits were identified via GWAS with Bonferroni correction (*P* ≤ 3.42e-06), including 16 for TL, 18 for SA, 27 for NR, and 10 for AD (Table S7, Fig. 4). Notably, eight significant SNPs were consistently identified by two of the models. Additionally, ten significant SNPs were simultaneously detected for TL and SA. The intervals of 100 kb surrounding the 71 GWAS signals corresponded to 98 candidate genes associated with rooting traits (Table S8). Subsequently, transcriptomic sequencing of genotypes with contrasting rooting abilities was conducted to validate the gene associations. A total of 29 348 differentially expressed genes were identified, including 13 954 upregulated genes and 15 394 downregulated genes (Table S9, Fig. S5). Approximately, 21% of the candidate genes identified by GWAS showed differential expression between accessions with strong and weak rooting ability.

For TL, several GWAS signals were identified on chromosomes 1, 7, 9, 14, 15, and 17, with thirty-two candidate genes located in their vicinity. On chromosome 7, a locus (Chr7_53918477) within an intron of *evm.model.scaffold_1748.405.1*, homologous to *MPK9*, is involved in reactive oxygen species-mediated ABA signaling [17]. Transcriptome and quantitative real-time PCR (qRT-PCR) analyses revealed significant upregulation of this gene in chrysanthemums with strong rooting capacity (Table S8; Fig. S6a). Haplotype analysis revealed that SNP variants at this site increase root length, and accessions carrying the SNP variant are predominantly of the PGC cultivation type (Fig. S6a). Another locus (Chr1_272173254) was positioned downstream of *evm.model.scaffold_1072.140* (*FER*) and near *evm.model.scaffold_1072.14* (*MDS2*), both of which are implicated in root growth regulation under environmental stress [28, 30]. The GWAS signals Chr14_135335406, Chr15_287247056, and Chr17_217576708 were located near the *evm.model.scaffold_1314.59* (*FAB1A*), *evm.model.scaffold_4404.215* (*PAP28*), and *evm.model.scaffold_10191.40* (*ALS1*), respectively. These genes are known to regulate root growth, phosphorus acquisition, and aluminum detoxification in model plants [6, 13, 16, 24].

For SA, GWAS signals were mapped on chromosomes 2, 5, 7, 9, 14, and 15, yielding twenty-nine candidate genes in nearby genomic regions. On chromosome 7, four candidate genes were identified through the GWAS signal Chr7_201791696 (Fig. 4b; Table S6). Among these, *evm.model.scaffold_9340.590*, *evm.model.scaffold_9340.591*, and *evm.model.scaffold_9340.592* belong to group II of the Gretchen Hagens 3 (GH3) family, which plays a crucial role in auxin inactivation and influences various stages of lateral root development [43]. Remarkably, *evm.model.scaffold_9340.589*, an orthologue of *NRAMP3* essential for iron mobilization in germinating seeds in *Arabidopsis* [23], overlapped with a domestication sweep (Fig. 4c) and showed downregulated expression in the accession with strong root growth ability (Table S8; Fig. 4d). Haplotype analysis revealed that SNP variants at this site contribute to increased root surface area. In cultivars carrying the variant allele (CA genotype), the SCC type is more prevalent than the TC type. In contrast, in noncarrier cultivars (CC genotype), the TC type dominates. This pattern suggests potential selection for rooting ability in chrysanthemum breeding (Fig. 4d).

For AD, GWAS signals were detected on chromosomes 2, 6, 12, 14, and 16, encompassing seventeen candidate genes. A significant SNP (Chr2_163240747), detected in both models, is located near *evm.model.scaffold_481.126* (Fig. 4, e), an orthologue of *Arabidopsis CML20*, which is involved in ABA regulation of guard cell channels and the accumulation of stress-responsive transcripts [45]. This gene is upregulated in chrysanthemum accessions with strong rooting ability (Fig. S6b). Haplotype analysis revealed that SNP variants at this site result in a reduced root diameter, with no clear preference for cultivated types between the two genotypes (GG and GA) (Fig. S6b).

For NR, GWAS signals were found on chromosomes 2, 3, 4, 5, 7, 8, 10, 11, 16, 17, and 23, leading to the identification of sixty-two candidate genes. On chromosome 5, a signal was located proximal to *evm.model.scaffold_1631.63*, orthologous to *SPL14*, whose mutants in rice display insensitivity of root elongation response to nitrate supply [37]. This gene showed differential expression between accessions with strong and weak rooting ability, highlighting its role in the rooting of chrysanthemum cuttings (Table S8). The GWAS signal Chr11_187947859 is located in an exonic region of the gene *evm.model.scaffold_830.39_evm.model.scaffold_830.40*, an orthologue of *UFL1* with unknown function, which overlaps with a domestication sweep. Interestingly, *evm.model.scaffold_1113.144* near the GWAS signal Chr23_209923210 falls within a putative domestication sweep (Fig. 4g) and is upregulated in chrysanthemum accessions with strong rooting ability (Fig. 4h). It is orthologous to *Arabidopsis SKS5* and plays a role in root growth and cotyledon vascular formation in *A. thaliana* [5]. Haplotype analysis showed that SNP variation at this locus is associated with increased roots. The TC type is primarily found in accessions with the homozygous non-variant genotype (AA), whereas all accessions with the homozygous variant genotype (GG) belong to the SCC cultivation type (Fig. 4h).

### Co-expression network and pathway analysis

**Figure 5:**
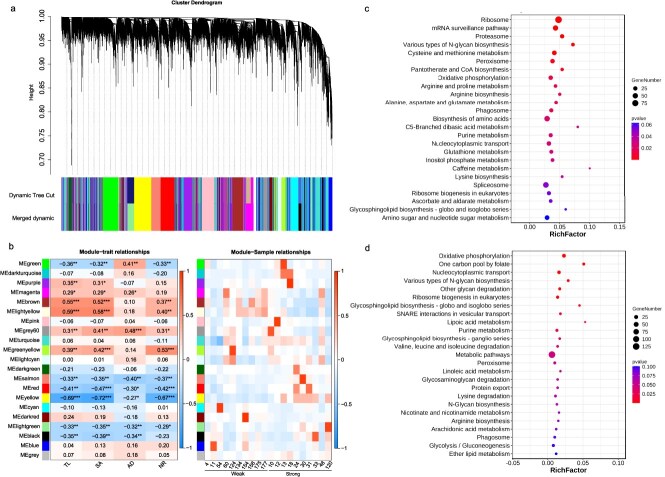
Clustering of module eigengenes and correlations between gene module and rooting traits in chrysanthemum. (a) Cluster dendrogram of genes based on a co-expression network analysis. (b) Correlation heatmap illustrating the associations between WGCNA modules and rooting traits, as well as between samples with contrasting rooting abilities and qualities. Asterisks denote significant differences as determined by the Student’s *t*-test (^***^P < 0.001, ^*^  *P* < 0.05). (c, d) KEGG pathway analysis for the genes in the yellow (c) and lightyellow modules (d), where circle size represents the number of genes, and color changes indicate corresponding changes in p-values.

To obtain a deeper understanding of the relationship between rooting traits and gene expression in chrysanthemum, a weighted gene co-expression network analysis (WGCNA) was performed using the expression data from 41 625 genes across all samples. A total of 21 gene co-expression modules were identified, with the number of genes in each module ranging from 501 to 6603 (Fig. 5a). Notably, yellow and lightyellow modules displayed distinct expression patterns between accessions with strong and weak rooting ability (Fig. 5b). KEGG enrichment analysis revealed that both yellow and lightyellow modules were significantly enriched in pathways related to oxidative phosphorylation, ribosome biosynthesis, peroxisome synthesis, and various types of N-glycan biosynthesis (Fig. 5c and d). Carbohydrates like glucose provide essential energy and carbon sources for adventitious root formation, while antioxidant enzymes such as peroxidase help mitigate oxidative stress and enhance cell activity during plant rooting. Additionally, the yellow module was notably enriched in pathways related to cysteine and methionine metabolism and arginine and proline metabolism, which are crucial for various physiological and biochemical processes in plant growth and development.

Within the yellow and lightyellow modules, 47 and 38 hub genes were identified, respectively (Table S10; Fig. S7a and b). Several are known to be involved in root development, including *evm.model.scaffold_610.4*, a homolog of *SDG34*, and *evm.model.scaffold_1186.15*, a homolog of *MTM1*, both of which play roles in root growth responses [4, 15], as well as *evm.model.scaffold_1787.281*, a homolog of *DOF2.1*, which regulates radial root expansion [55]. RNA-seq data indicated that 68 hub genes exhibited differential expression in chrysanthemums with contrasting rooting ability (Table S9; Fig. S7c). Integrating candidate genes from GWAS with those identified in trait-correlated WGCNA modules yielded 14 overlapping candidates (Table S8). Among these, *evm.model.scaffold_9340.589*—a homolog of *NRAMP3*—stands out as it is not only differentially expressed and under selection but also emerged as a GWAS candidate located within the lightgreen module. To strengthen the connection between this gene and the GWAS signal at Chr7_201791696, we examined the association of the SNP genotype with the expression of *evm.model.scaffold_9340.589*. An analysis of the RNA-seq cohort revealed significantly higher expression in CC genotypes compared to CA (Wilcoxon rank-sum test, *P* = 0.0018; Fig. S8). Consistent with the gene’s inhibitory role in rooting, CC accessions displayed lower mean rooting ability than CA accessions (0.38 vs. 0.60; Table S1).

### Functional validation of *CmNRAMP3*

**Figure 6:**
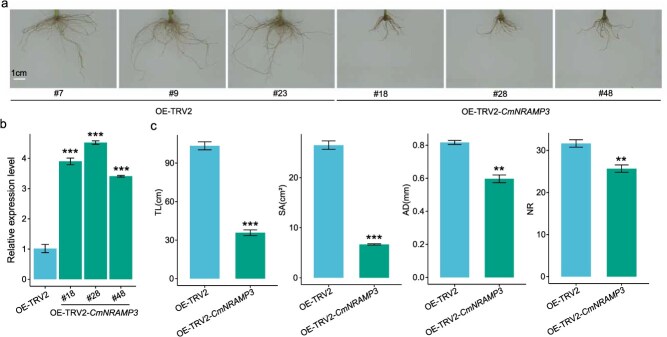
Phenotypic analysis of *CmNRAMP3* transiently transformed plants in chrysanthemum cultivar ‘Jinba’. Phenotypic comparison of the empty vector control (OE-TRV2) and transient *CmNRAMP3* overexpression (OE-TRV2-*CmNRAMP3*). Scale bar =1 cm. (b) RT-qPCR analysis showing relative *CmNRAMP3* expression in transient overexpression lines versus control. (c) Quantitative comparison of rooting traits: TL, SA, AD, and NR between control and overexpression lines. Data are presented as mean ± standard deviation (n = 3). Statistical significance was determined using Student’s *t*-test (^**^P < 0.01, ^***^P < 0.001).

To investigate the role of the candidate gene *CmNRAMP3* in the regulation of rooting ability in chrysanthemum, an overexpression vector (OE-TRV2-*CmNRAMP3*) was constructed and transiently introduced into the cultivar ‘Jinba’ via *Agrobacterium*-mediated infiltration (Fig. 6a). RT-qPCR analysis confirmed that the expression of *CmNRAMP3* in overexpression lines increased by approximately 3.9-fold compared to the empty vector control, indicating effective upregulation of the target gene within a short period (Fig. 6b). Phenotypic assessments revealed that overexpression of *CmNRAMP3* significantly suppressed four key rooting traits—TL, SA, AD, and NR—relative to the control (Fig. 6a and c). These findings support that *CmNRAMP3* functions as a negative regulator of root system development in chrysanthemum.

## Discussion

Cutting is the primary propagation method in chrysanthemum production. In modern commercial chrysanthemum production, advanced techniques such as plant growth regulators and environmental control are employed to enhance the root quality of cuttings. However, significant variation in rooting capacity among different varieties poses a major challenge to achieving consistency in large-scale production. Economic losses due to low rooting efficiency remain an ongoing issue for the chrysanthemum industry [12]. Therefore, understanding the genetic architecture of rooting ability in chrysanthemum cuttings is essential to breeding varieties with robust rooting capabilities. Germplasm resources are foundational for breeding innovation. In this study, we evaluated the rooting abilities of 188 chrysanthemum genotypes through a root imaging system, focusing on root length, area, diameter, count, and weight. By integrating PCA and membership function approaches, we identified 34 germplasms exhibiting strong rooting abilities, predominantly of the SCC type. These germplasms provide valuable resources for investigating the genetic regulation of rooting in chrysanthemum cuttings.

Faster rooting speed and stronger root systems are key selection targets in modern chrysanthemum breeding. Phenotypic analysis revealed significant differences in rooting abilities across chrysanthemum types, with PGC and SCC outperforming TC. Population structure analysis grouped most SCC, TC, and PGC into three distinct genetic clusters: G1, G2, and G3, respectively. Compared with G2 (predominantly TC), the lower genomic diversity observed in G1 (primarily SCC) and G3 (mainly PGC) reflects their more recent and specialized breeding histories. As highly commercialized varieties dependent on clonal propagation, SCC and PGC require well-developed rooting capacity to ensure high productivity. In addition, TC displays the greatest diversity in plant architecture, flower form, and coloration. SCC and PGC, on the other hand, exhibit more uniform plant architecture and simpler floral morphologies: SCC are distinguished by upright growth and neatly arranged flowers, whereas PGC typically feature a compact, rounded plant type with smaller blooms. Consequently, SCC and PGC have been subjected to intensive directional selection for traits such as plant architecture, flower type and color, and rooting efficiency. The sustained selection has driven selective sweeps and genetic hitchhiking, further eroding genetic diversity [9]. Here, selective sweep analysis identified 534 genomic regions potentially under positive selection for rooting traits, harboring genes involved in cellulose biosynthesis, metabolic processes, signal transduction regulation, and root development genes (e.g., *RGL1*, *SRS5*, *ARF2*). These regions provide essential biological and evolutionary context for subsequent GWAS analyses, serving as auxiliary validation for significant SNPs identified in GWAS. GWAS, a powerful tool for elucidating the genetic basis of complex traits, has recently yielded important insights into key horticultural traits in chrysanthemum [8, 35, 53]. Building on this foundation, we performed GWAS for rooting traits using the GLM and MLM models in TASSEL and the MLM model in EMMAX. A total of 71 significantly associated SNPs were identified, including seven consistently detected across different models. These consistently detected loci suggest their potential stability across analytical methods, providing valuable candidate markers for further genetic analysis of rooting traits. The stringent thresholds of the MLM model resulted in the detection of only a few significant loci across four traits, likely increasing the false-negative rate and excluding genuine associations. By contrast, the multimodel approach reduced false positives caused by population structure and enhanced detection reliability, aligning with previous findings that SNPs replicated across methods tend to be more accurate [18]. Together, the integration of selective sweep analysis and GWAS highlights robust candidate loci and genomic regions underlying chrysanthemum rooting ability, providing valuable targets for future genetic improvement.

The highly complex chromosomal architecture of chrysanthemum, with polyploidy levels ranging from 2*n* = 18 to 2*n* = 8*x* = 72 and a genome size exceeding 8 Gb, has long hindered the precise identification of causal genes underlying horticultural traits. By integrating selective sweep analysis, GWAS, transcriptome analysis, and WGCNA, our study overcame these challenges and uncovered key regulators of rooting traits. A consistent theme emerging from our results is the central involvement of plant hormone pathways. Candidate genes related to gibberellin, ethylene, abscisic acid, and auxin signaling were enriched near selective sweeps and GWAS loci, highlighting the pivotal role of hormonal crosstalk in adventitious root formation, a process emphasized in diverse species [3, 31]. A particularly noteworthy candidate gene is *evm.model.scafffold_9340.589*, homologous to *NRAMP3*, and its corresponding SNP targets different haplotypes in TC and SCC, indicating that it has undergone strong artificial selection. qRT-PCR validation revealed significant differences in the relative expression levels of this gene between genotypes with contrasting rooting abilities, further supporting the GWAS findings. Moreover, *CmNRAMP3* overexpression weakened rooting performance in transiently transformed cuttings, providing direct functional evidence of its involvement. This result confirms that *CmNRAMP3* acts as a negative regulator of root development in chrysanthemum. Importantly, this gene is located in the lightgreen module identified by WGCNA, which was significantly correlated with TL, SA, and NR, further strengthening its candidacy. Previous research has demonstrated that *NRAMP3* is critical in root adaptation to low-iron environments, enhancing root vitality and overall plant health [27]. Additionally, WGCNA revealed that yellow and lightyellow were strongly correlated with TL, SA, and NR. Notably, most hub genes identified in the yellow and lightyellow modules by WGCNA were not identified in the GWAS results. This discrepancy is expected, as the two approaches capture distinct aspects of trait-gene relationships: GWAS detects loci statistically associated with phenotypic variation, while WGCNA highlights genes with high co-expression connectivity, reflecting transcriptional co-regulation. Genes identified by GWAS may act as upstream regulators, show condition-specific expression, or function through post-transcriptional mechanisms—features that could contribute to their low connectivity within the co-expression network. In contrast, some hub genes may be absent from GWAS due to weak linkage disequilibrium with trait-associated SNPs [33]. Therefore, integrating GWAS and WGCNA provides complementary insights, increasing confidence in overlapping candidates and broadening the set of plausible genes for functional validation.

In summary, we have elucidated the genomic signatures subject to artificial selection for root capability in vegetatively propagated chrysanthemums during the breeding process. The identified SNP variants and candidate genes provide novel insights into the genetic basis governing the rooting ability of chrysanthemum cuttings and offer a promising gene pool for the development of superior cultivars through molecular breeding strategies. Furthermore, our research serves as a valuable example for improving the rooting capability of other vegetatively propagated crops.

## Materials and methods

### Plant materials

A total of 188 representative chrysanthemum genotypes from diverse origins with no direct genetic relationships were used, including 58 SCC, 58 PGC, 59 TC, and 13 WC. All materials were preserved at the Chrysanthemum Germplasm Resources Preserving Center, Nanjing Agricultural University, China. Experiments were conducted in May 2017 (Y1) and May 2020 (Y2) within a film greenhouse. Tender top shoots (6–8 cm) from healthy mother plants were collected as cuttings, placed in a 105-well seedling tray with perlite, and inserted to a depth of 2–3 cm. A completely randomized block design was employed, with three replicates of five plants each.

### Assessment of rooting ability in cuttings

After 20 days of rooting, the plants were removed from trays, and the perlite adhering to the roots was thoroughly washed off. The total root length (TL), projected root area (PA), root surface area (SA), average root diameter (AD), root volume (V), number of root tips (NT), and maximum total root length (MTL) were measured using the WinRHIZO root image analysis system (Regent Instruments Inc., Québec, Canada). The underground fresh weight (UFW) was recorded using an analytical balance, and the underground dry weight (UDW) was measured after oven-drying at 80°C for 24 h. The number of roots (NR) was manually counted, and the average root length (AL) was calculated by dividing the TL by NR.

### Data processing and analysis

All statistical analyses were conducted in R version 4.0.4 (https://www.r-project.org/). The ‘psych’ package was used for normality tests, descriptive statistics, and principal component analysis (PCA), and. The ‘lme4’ package for estimating best linear unbiased estimates (BLUEs). Broad-sense heritability was calculated using the following formula:${h}_B^2={V}_g/\left({V}_g+{V}_{ge}/\mathrm{l}+{V}_e/\mathrm{rl}\right)$, where${V}_g$, ${V}_e$, and ${V}_{ge}$ represent the genotype variance, environmental variance, and genotype-environment interaction variance (G × E), respectively, and l and r represent the number of environments and replicates, respectively. PCA was performed on the BLUE of the 11 rooting traits, with the number of PCs (n) determined by the cumulative variance and eigenvalues. Component weights was calculated as *W* = contribution rate/cumulative contribution rate. Membership function value was computed as *U_m_* = (*CI* – *CI_min_*)/(*CI_max_* – *CI_min_*), and the comprehensive rooting ability score was calculated as *D* = ∑ *U* · *W*.

### Genotyping and population analysis

The genotyping data for the 188 chrysanthemum accessions were obtained from a previously sequenced panel of 346 chrysanthemum germplasm using genotyping-by-sequencing (GBS) technology [53]. The sequencing was conducted at an average depth of 11.59×, covering approximately 6.26% of the genome. SNP calling was performed using the genome of chrysanthemum ‘Zhongshan Zigui’ [34] as the reference. To ensure data quality, raw SNPs were filtered based on two criteria: minor allele frequency (MAF) < 0.05 and a missing data rate < 20%, resulting in 370 562 high-quality SNPs for downstream analysis.

Population structure analysis was conducted using the Admixture software [1], with the predefined number of populations (K) ranging from 1 to 10. The optimal K value was determined based on the smallest cross-validation error (*CV*). A phylogenetic tree was constructed using the neighbor-joining (NJ) method in MEGA11 [39], with 1000 bootstrap replicates, and subsequently refined using the ITOL website (https://itol.embl.de/). PCA was performed using GCTA software [48], and the kinship matrix for the population was calculated.

### Detection of selective sweeps

To evaluate genetic diversity related to rooting ability among chrysanthemum subgroups, we calculated genetic differentiation (*F_ST_*) between subgroups with different rooting performance, and nucleotide diversity (π) within each subgroup. These analyses were conducted using VCFtools [10] with a sliding-window approach (100 kb window, 10 kb step). To pinpoint genomic regions potentially under selection during breeding for enhanced rooting ability, we identified candidate selection regions as those falling within the top 5% of both *F_ST_* (between TC and SCC) and the π ratio (π_TC_/π_SCC_) distributions. Candidate genes located within these selection regions were annotated using the Gene Ontology (GO) database (http://geneontology.org). GO enrichment analysis of annotated genes was conducted with the OmicShare tools (https://www.omicshare.com/tools), an online platform for data analysis. Enrichment significance was assessed using a hypergeometric test with *P* values adjusted by FDR, and GO terms with corrected *P ≤* 0.05 were considered significantly enriched.

### GWAS and annotation of candidate genes

Association analysis for four key rooting traits (TL, SA, AD, and NR) was performed using the GLM and MLM models in TASSEL [2] and the MLM model in EMMAX [19]. For the GLM, the population structure Q matrix was used as a covariate, while for MLM and EMMAX, both the Q matrix and kinship K matrix were used as covariates. The effective number of SNPs (*Me =* 292 493) was calculated using the GEC (Genetic Type I error calculator) application [25], and a significance threshold was determined using the Bonferroni correction (*P ≤* 1/292 493 = 3.42e-06). The association results were visualized using the ‘CMplot’ R package [49].

Candidate genes were explored within a 50-kb interval upstream and downstream of the genomic locations of significantly associated SNP loci. The potential functions of these candidate genes were extracted using the chrysanthemum ‘Zhongshan Zigui’ reference genome [34]. Further annotation of the candidate genes was performed using multiple databases, including the *Arabidopsis thaliana* genomic database (www.arabidopsis.org), Swiss-Prot (www.uniprot.org), and relevant publicly available literature.

### Transcriptome sequencing and co-expression module analysis

Based on comprehensive rooting ability scores (*D*) of 188 chrysanthemum genotypes, 10 strong-rooting (mean *D* = 0.68) and 10 weak-rooting (mean *D* = 0.25) varieties were selected (Table S1). Root apical meristems were sampled at 20 days post-cutting, with three biological replicates for each variety. Total RNA was extracted, and 60 libraries were sequenced on the Illumina platform. Clean reads were aligned to the reference genome with Hisat2 [20], and gene expression levels were quantified using HTSeq 2.0 [29] to generate an Fragments Per Kilobase of exon model per Million (FPKM mapped fragments) expression matrix. Differentially expressed genes (DEGs) were analyzed using the R package ‘DESeq2’ with the criteria of adjusted *P*-value (padj) ≤ 0.05 and |log_2_ (FoldChange)| ≥ 1. Genes with log_2_ (FoldChange) ≥ 1 were considered upregulated in strong-rooting genotypes relative to weak-rooting genotypes, whereas those with log_2_ (FoldChange) ≤ −1 were considered downregulated.

WGCNA was conducted using the R package ‘WGCNA’ [22] on the top 30% median absolute deviation (MAD) genes. An optimal soft-threshold was chosen to ensure scale-free topology, and co-expression modules were constructed and merged at a similarity exceeding 0.7. Module-trait correlations were calculated for four key rooting traits to identify trait-associated modules. KEGG enrichment analysis of the genes in the module of interest was performed using OmicShare tools (hypergeometric test, FDR ≤ 0.05). Hub genes were defined by |gene significance| (|GS|) > 0.2 and |module membership| (|MM|) > 0.8, visualized using OmicShare tools. The unconnected hub genes were excluded from the final network.

### Quantitative real-time PCR

Total RNA extracted from apical meristems of roots at 20 days post-cutting from two cultivars displaying contrasting rooting capacities: ‘Nannong Hengyun’ (code: 46, strong) and ‘Chixian Jinzhu’ (code: 124, weak). The qRT-PCR was performed using TB Green Premix Ex Taq II (TaKaRa, Japan) with three biological replicates included for each sample. *EF1α* was used as the reference gene, and the relative expression levels of the candidate genes were calculated using the 2^−ΔΔCT^ method [26]. Primers sequences were listed in Table S2.

### Cloning and overexpression transient transformation of *CmNRAMP3*

Full-length primers for *CmNRAMP3* were designed based on the chrysanthemum ‘Zhongshan Zigui’ reference genome (Table S2). The cDNA from the cultivar ‘Jinba’ was used as the template for gene cloning, and the amplified fragment was inserted into the OE-TRV2 vectors to generate the recombinant construct (OE-TRV2-*CmNRAMP3*). The construct was introduced into *Agrobacterium tumefaciens* GV3101 and subsequently used to infect cuttings of ‘Jinba’. Newly developed leaves were collected for genomic DNA extraction to confirm positive transformants and for RNA extraction to evaluate *CmNRAMP3* expression by RT-qPCR. Rooting performance was assessed by measuring four key traits (TL, SA, AD, and NR) in successfully transformed lines.

## Supplementary Material

Web_Material_uhaf289

## Data Availability

The GBS data utilized in this study are publicly available in the National Center for Biotechnology Information (NCBI) Sequence Read Archive (SRA) under the BioProject accession number PRJNA1004079. All other data generated or analyzed in this study are provided within the article and its supplementary materials.
