# Supplementary material for: Elucidation of the genetic basis underlying rooting ability in vegetatively propagated chrysanthemum
Source: Hortic Res. 2025 Nov 3;13(2):uhaf289. doi: 10.1093/hr/uhaf289 (PMC12903453; doi:10.1093/hr/uhaf289)
Supplement: Web_Material_uhaf289 [file web_material_uhaf289.zip › Supplementary Figures.docx]

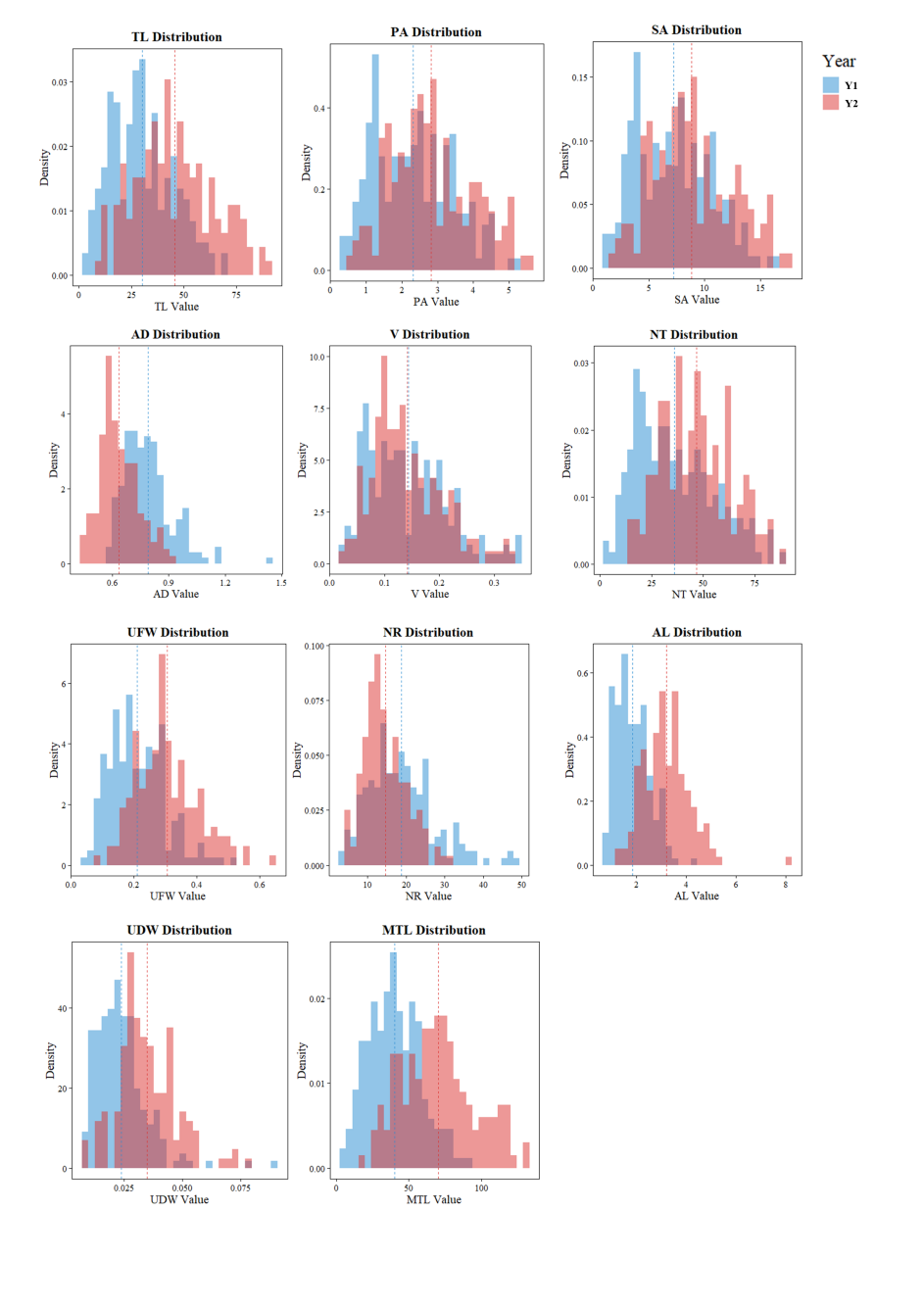


Fig. S1 Frequency distribution of 11 rooting traits of 188 chrysanthemum genotypes in Y1 and Y2.


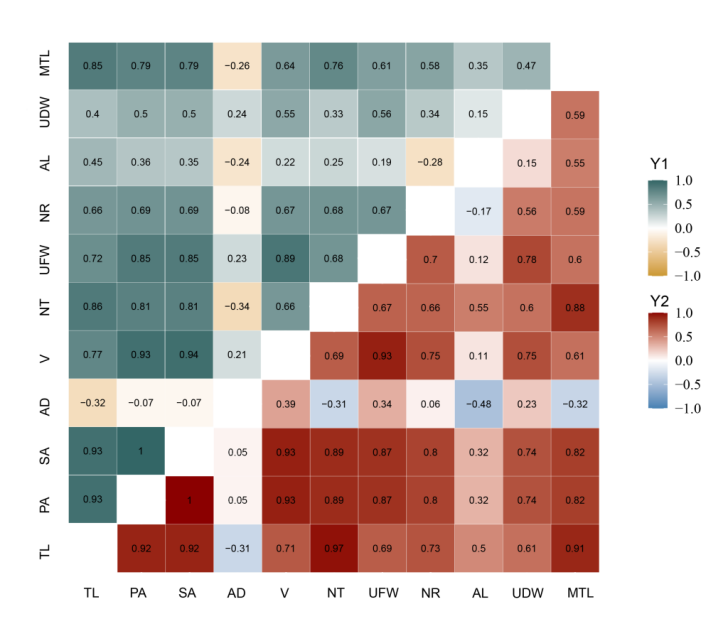


Fig. S2 The heat map of Pearson’s correlation among the 11 rooting traits of 188 chrysanthemum genotypes in Y1 (upper left square) and Y2 (lower right square).


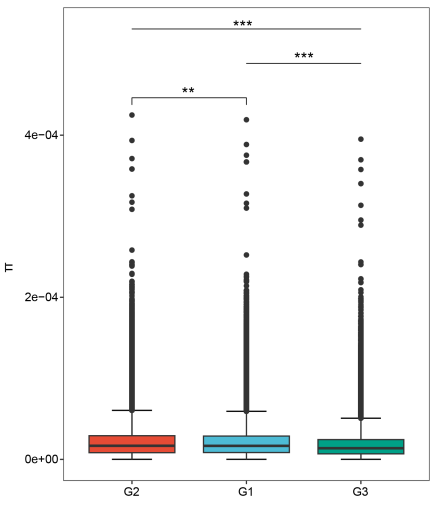


Fig. S3 Comparison of π among three subpopulations: G1, G2, and G3. Asterisks indicates significant difference as analyzed by Student’s t-test (*** *P* < 0.001, and ***P* < 0.01).


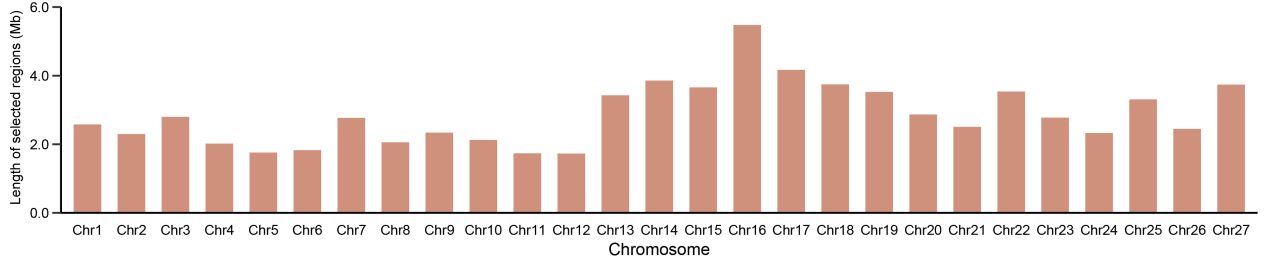


Fig. S4 Distribution of selected-region length (Mb) across chromosomes, identified using both *F_ST_* and π ratios metrics.


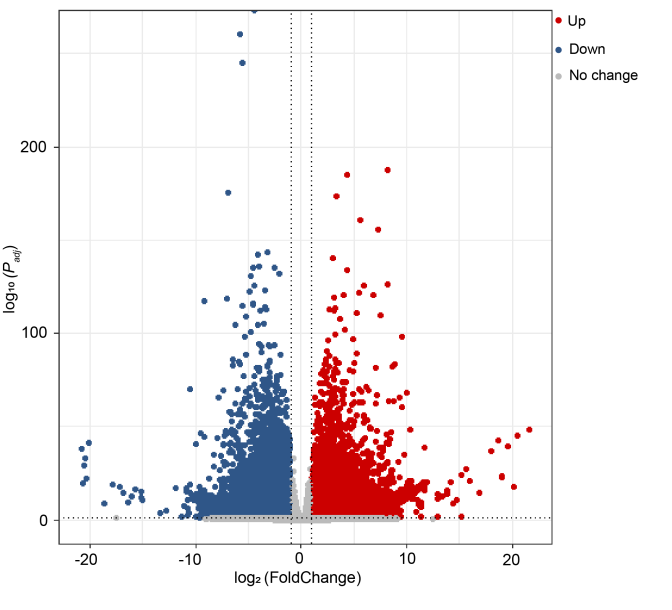


Fig. S5 Volcano plot of gene expression differences between different chrysanthemum genotypes with contrasting rooting abilities.


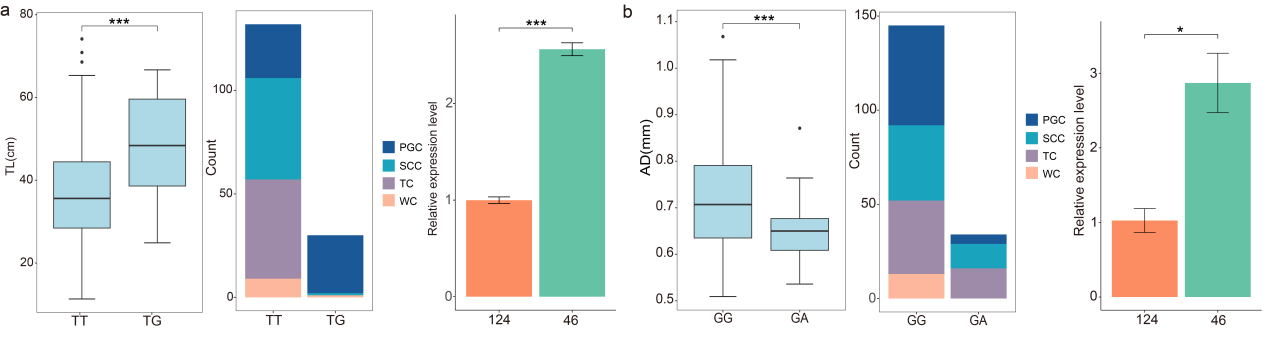


Fig. S6 Expression patterns of candidate genes and haplotype analysis of corresponding SNP. (a) Haplotype analysis and distribution of TL across different cultivation types at Chr7_53918477, along with a comparison of *MPK9* expression levels between accessions with weak (124) and strong (46) rooting abilities. (b) Haplotype analysis and the distribution of AD across different cultivation types at Chr2_163240747, along with a comparison of *CML20* expression levels between accessions with weak (124) and strong (46) rooting abilities. Asterisks denote significant differences as determined by Student’s t-test (****P* < 0.001, * *P* < 0.05).


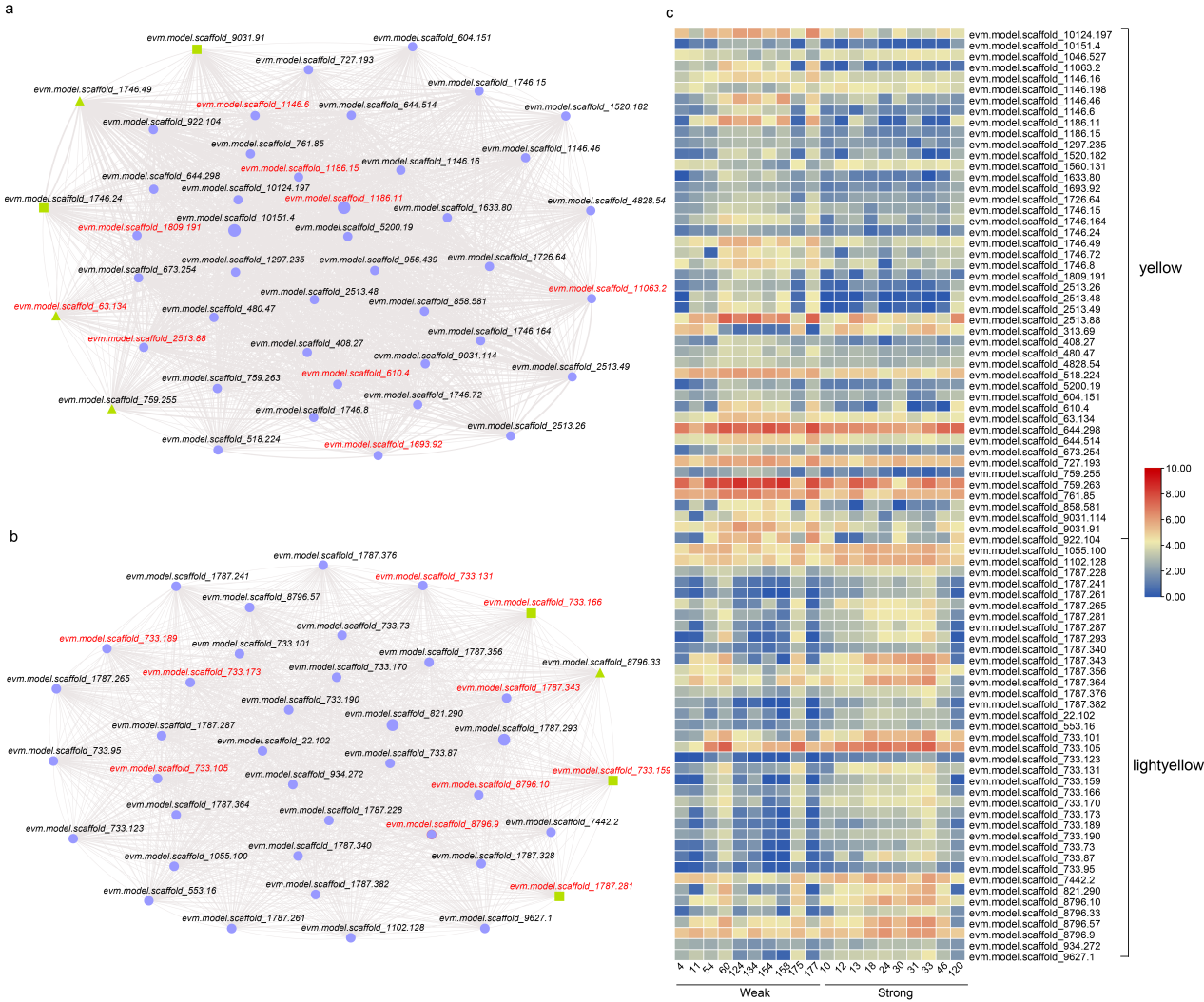


Fig. S7 Regulatory network and expression patterns of hub genes in yellow and lightyellow module. (a, b) Network maps for hub genes belonging to the yellow (a) and lightyellow (b) module. Nodes of different shapes represent hub genes, with squares for transcription factors and triangles for protein kinases. Connections between nodes indicate the degree of connectivity, and genes marked in red are differential expressed in RNA-seq analysis. (c) Heatmap showing expression patterns of hub gene in yellow and lightyellow module.


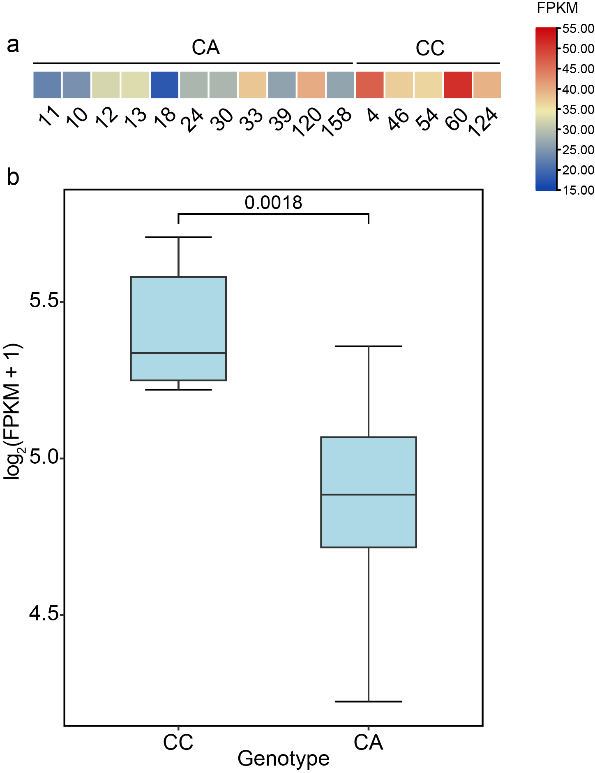


Fig. S8. Expression variation of *evm.model.scaffold_9340.589* associated with the significant SNP Chr7_201791696. (a) Heatmap of FPKM values for accessions carrying different genotypes (CA vs. CC). Each block represents an individual sample, with colors indicating expression levels. (b) Boxplot showing significantly higher expression in CC genotypes compared with CA genotypes (Wilcoxon rank-sum test, *P* = 0.0018).
